# Supplementary material for: The interactome of intact mitochondria by cross-linking mass spectrometry provides evidence for coexisting respiratory supercomplexes
Source: Mol Cell Proteomics. 2017 Dec 8;17(2):216–32. doi: 10.1074/mcp.RA117.000470 (PMC5795388; doi:10.1074/mcp.RA117.000470)

LVPLKETIK
VRVDEYDYSKPIQGQQK

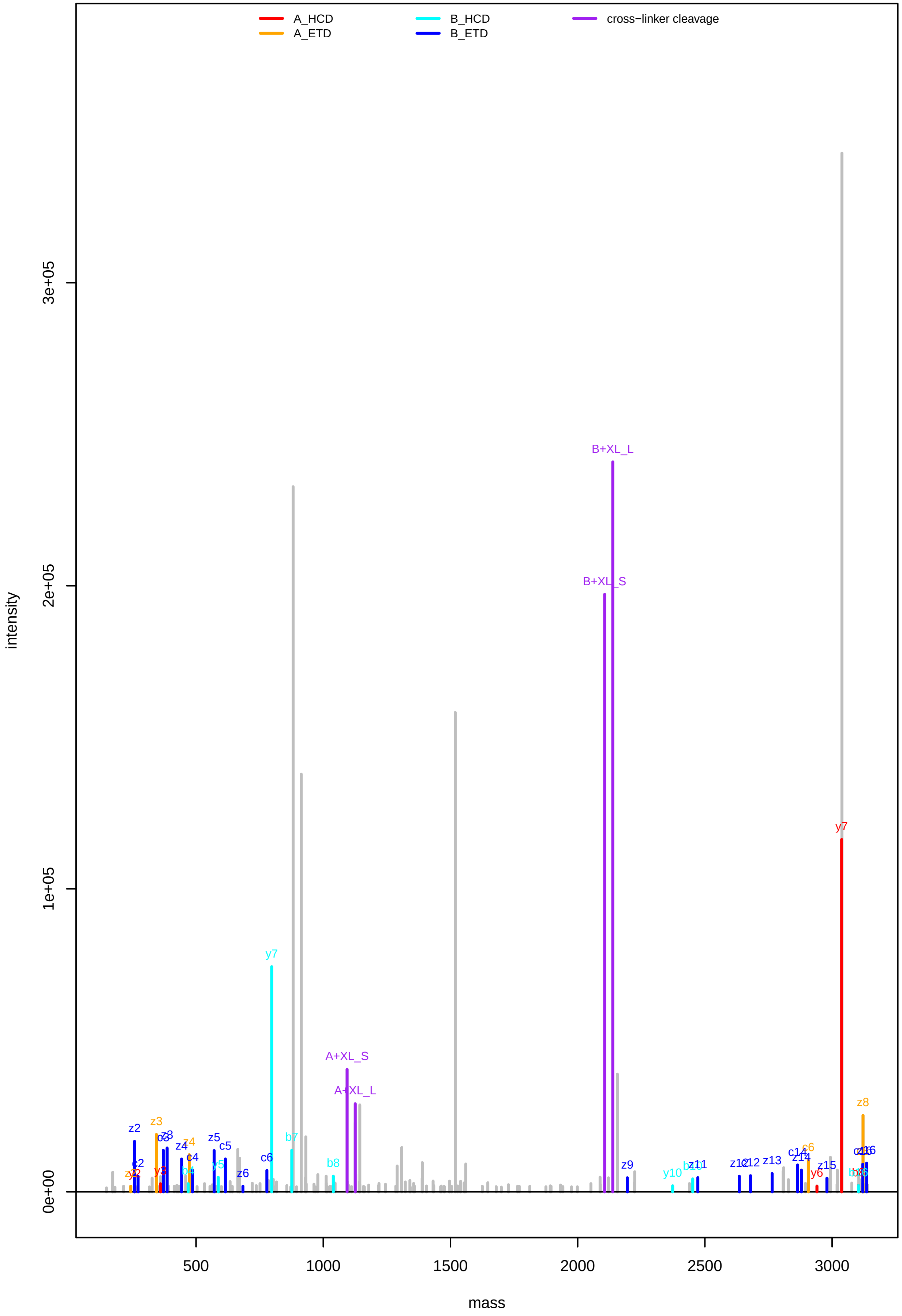

LVPLKETIK+XL_S

LVPLKETIK+XL_L

VRVDEYDYSKPIQGQQK+XL_S

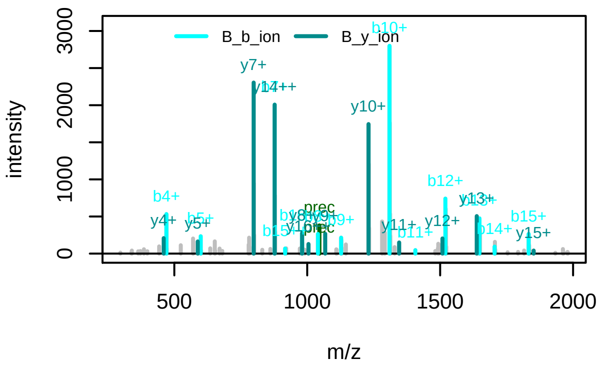

VRVDEYDYSKPIQGQQK+XL_L

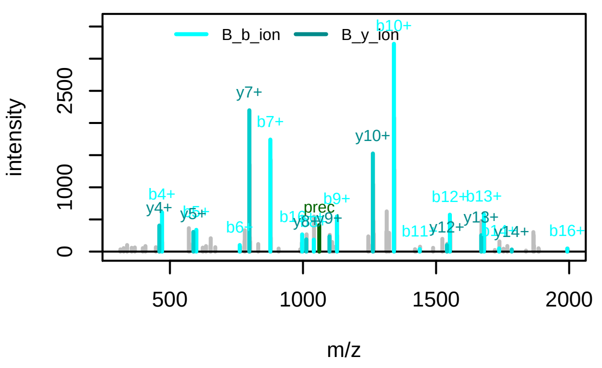

Supplement: Supplemental Data [file supp_RA117.000470_133922_0_supp_23978_fzffwf.zip › spectra_annotation/mito_spectra_annotation/70-1-4-1-10-1.pdf]
